# Mycosis fungoides and Sézary syndrome: clinical presentation, diagnosis, staging, and therapeutic management

**DOI:** 10.3389/fonc.2023.1141108

**Published:** 2023-04-14

**Authors:** Denis Miyashiro, José Antonio Sanches

**Affiliations:** Division of Clinical Dermatology, Hospital das Clínicas, University of São Paulo Medical School, São Paulo, Brazil

**Keywords:** mycosis fundgoides, Sézary syndrome, cutaneous T cell lymphoma, histopathology, prognosis, treatment

## Abstract

Mycosis fungoides (MF) and Sézary syndrome (SS) are cutaneous T-cell lymphomas. MF is the most common cutaneous lymphoma, and it is classified into classic Alibert-Bazin MF, folliculotropic MF, pagetoid reticulosis, and granulomatous slack skin, each with characteristic clinical presentation, histopathological findings, and distinct clinical behaviors. SS is an aggressive leukemic variant of cutaneous lymphoma, and it is characterized by erythroderma, lymphadenopathy, and peripheral blood involvement by malignant cells. There is a wide range of dermatological manifestations of MF/SS, and prompt recognition is essential for early diagnosis. Skin biopsy for histopathology and immunohistochemical analysis is imperative to confirm the diagnosis of MF/SS. Histopathology may also provide information that may influence prognosis and treatment. Staging follows the TNMB system. Besides advanced stage, other factors associated with poorer prognosis are advanced age, male gender, folliculotropism in histopathology of patients with infiltrated plaques and tumors in the head and neck region, large cell transformation, and elevated lactate dehydrogenase. Treatment is divided into skin-directed therapies (topical treatments, phototherapy, radiotherapy), and systemic therapies (biological response modifiers, targeted therapies, chemotherapy). Allogeneic bone marrow transplantation and extracorporeal photopheresis are other treatment modalities used in selected cases. This review discusses the main clinical characteristics, the histopathological/immunohistochemical findings, the staging system, and the therapeutic management of MF/SS.

## 1 Introduction

Extranodal lymphomas account for approximately 30% of non-Hodgkin lymphomas (1). The skin is the second most involved organ, after the gastrointestinal tract (2). Primary cutaneous lymphomas are classified into cutaneous NK/T-cell lymphomas (CTCL) and cutaneous B-cell lymphomas (CBCL), by the World Health Organization (WHO) and the European Organization for Research and Treatment of Cancer (EORTC). The CTCLs represent approximately 70% to 82% of all primary cutaneous lymphomas. Mycosis fungoides (MF) is the most common CTCL, representing almost 50% of all primary cutaneous lymphomas, and Sézary syndrome (SS) is a rare and aggressive leukemic disease (2–7).

MF was described in 1806 by Jean-Louis-Marc Alibert and, later, Pierre-Antoine-Ernest Bazin described the progression from patches (non-infiltrated lesions with erythema, scaling, and atrophy), to infiltrated plaques and tumors. Histologically, it is characterized by the proliferation of small and medium-sized epidermotropic CD4+ T-lymphocytes with convoluted nuclei (4).

SS was described by Albert Sézary and Yves Bouvrain in 1938 (8, 9). It is a leukemic variant of CTCL and occurs almost exclusively in adults. It is classically characterized by erythroderma, lymphadenopathy, and neoplastic cells in peripheral blood (3, 10).

In this review, we aim to describe the clinical presentation, diagnosis, staging system, and treatment currently available for MF and SS.

## 2 Epidemiology

It is estimated that primary cutaneous lymphomas have an annual incidence between 0.3 and 10.7 new cases per 1,000,000 individuals (1, 2, 5, 6). Approximately 70 to 82% of primary cutaneous lymphomas are CTCL, with annual incidences ranging between 3.4 and 7.7 per 1,000,000 individuals (2, 5, 6) and absolute predominance of MF and its variants and subtypes, which accounts for approximately 50% of the cases of cutaneous lymphomas (3, 4). Annual incidences of MF and SS range from 2.0 to 4.1 cases per 1,000,000 and 0.1 to 0.3 cases per 1,000,000, respectively (2, 5, 6). The incidence of cutaneous lymphomas is increasing, currently with an incidence three times higher than in the early 1980s (2).

MF typically affects adults with 50 to 60 years at diagnosis, and the disease is rarely described in childhood and young adults, except in the hypopigmented subtype of MF, with median age at diagnosis of 32 years (2, 11–15). Sézary syndrome (SS) is a rare and aggressive variant of CTCL that typically affect the elderly with a median age at diagnosis between 60 and 65 years (3, 4, 10, 13).

A systematic review and meta-analysis study (16) that analyzed North American (11, 17–22), European (23–26), Asian (12), and Australian (27) studies, included 6,279 patients with MF and SS and showed a predominance of males, accounting for 53% to 73% of the patients.

The analysis of the distribution of cutaneous lymphomas among different ethnicities is difficult due to the heterogeneity of this characteristic in different countries. An international and prospective multicenter study (Prospective Cutaneous Lymphoma International Prognostic Index - PROCLIPI study), which encompasses 19 countries from 6 continents, reported an absolute predominance of white patients (82.8%), reflecting a large number of North American and European centers (28). Data on the impact of ethnicity on disease behavior are conflicting due to the difficulty in the analysis of this parameter, however, there is evidence that black MF/SS patients are younger, have a female predominance, and are at higher risk of disease progression (28–30).

## 3 Pathogenesis

The pathogenesis of cutaneous lymphomas is not completely understood. It is believed that the chronic activation of T-cells by antigen-presenting cells leads to the gradual accumulation of mutations that culminate in the development of neoplastic cells. However, the triggering antigen for this chronic stimulation is unknown, and it could vary between patients (31). There is also the hypothesis that large plaque parapsoriasis and pityriasis lichenoides chronica would represent lymphocytic dyscrasias that could correspond to the link between polyclonal processes and MF (32, 33).

Bacterial, viral, fungal, and mycobacterial infections (34, 35), medications, and low vitamin D levels (36–39) have already been studied as the triggering factors in the origin of cutaneous lymphoproliferative processes. Analysis of regional grouping of patients with cutaneous lymphomas suggests exposure to unknown environmental factors and even occupational exposure (benzene and trichloroethylene) (40–43). However, none of these factors was consistently associated with the genesis of cutaneous lymphomas, and most of these studies are restricted to small series and case reports.

Studies analyzing the data of exome sequences of MF patients detected ultraviolet (UV) signatures in 10.8 to 57.6% of the mutational burden, suggesting that UV has a significant impact on the pathogenesis of MF (44). On the other hand, phototherapy with UVA or UVB is frequently used as a skin-directed therapy for MF/SS. The assessment of the interaction between UV and neoplastic cells and tumor microenvironment will add to the understanding of the real role of UV in MF/SS genesis and treatment.

MF and SS were considered the same disease for many years. However, the neoplastic cells of MF and SS have distinct immunophenotypic profiles. In MF, cells strongly express CCR4 and CLA, skin-homing receptors, and lack CCR7 and L-selectin, lymph nodes-homing receptors. This immunophenotype is typically observed in skin-resident memory T-cells. On the other hand, SS cells express CCR7 and L-selectin molecules, as well as CD27, a central memory T-cell marker, and they also strongly express CCR4 and other skin-homing receptors (CCR6, CCR10, CLA) (45). These findings suggest that MF and SS are distinct diseases, originating in different subtypes of T lymphocytes. However, the description of patches and plaques in MF patients that progress to a typical clinical picture of SS favors the hypothesis that there is a spectrum of manifestations encompassing the two entities. In addition to these observations, genetic and epigenetic studies demonstrate a great diversity in mutations and activated/inactivated signaling pathways in MF and SS (46, 47), and the same patient may have individually heterogeneous neoplastic cells (48). The genomic heterogeneity hinders the definition of whether MF and SS are part of the same disease, or if they are different diseases.

## 4 Clinical presentations

### 4.1 Mycosis fungoides

The WHO and EORTC classification of cutaneous lymphomas recognize the classic Alibert-Bazin type of MF and three variants: folliculotropic MF, pagetoid reticulosis, and granulomatous slack skin (GSS) (4). Besides the classic MF and the three variants, other clinicopathological subtypes of MF have been described: hypopigmented, poikilodermatous, erythrodermic, granulomatous, hyperpigmented, ichthyosiform, syringotropic, papular, purpuric, interstitial, pustular, bullous, verrucous, and psoriasiform MF. These other subtypes have clinical and pathological peculiarities but are included in the classic Alibert-Bazin MF group due to their similar prognostic features (14, 49–51).

Classic MF is the most frequent type, corresponding to 88.6%, followed by folliculotropic MF which corresponds to 11.4% of MF cases. Pagetoid reticulosis and granulomatous slack skin are extremely rare and represent less than 1% (4). Among the other MF subtypes included in the classic MF group, poikilodermatous MF is the most frequent (10% to 11%) followed by hypopigmented MF (3% to 10%) (13, 24).

Pruritus is the most frequent symptom, present in 80% of MF patients, and all patients with SS (13). Pruritus may be intense, especially in SS (10), and it is associated with significant worsening in quality of life (52).

Systemic symptoms are rarely seen in MF/SS. In a literature review study, Morris et al. evaluated 63 articles, including 505 patients with unusual findings in SS. Of these patients, just 8 patients (1.6%) had B symptoms, including fever, night sweats, and weight loss (53).

The time between symptom onset and diagnosis of MF varies between 2 years and 4.2 years (11, 54, 55). The PROCLIPI study observed that there is a diagnostic delay in early-stage MF, with median time between the first symptoms and diagnosis of 36 months (54). Cutaneous lymphomas are rare and are often misdiagnosed as eczematous processes, especially in the early stages of the disease. In addition, there is no single gold standard test that makes the diagnosis of MF or SS, but a set of clinical, histopathological, and molecular findings are needed, which contributes to the delay in the diagnosis.

#### 4.1.1 Classic Alibert-Bazin MF

**Figure 1:**
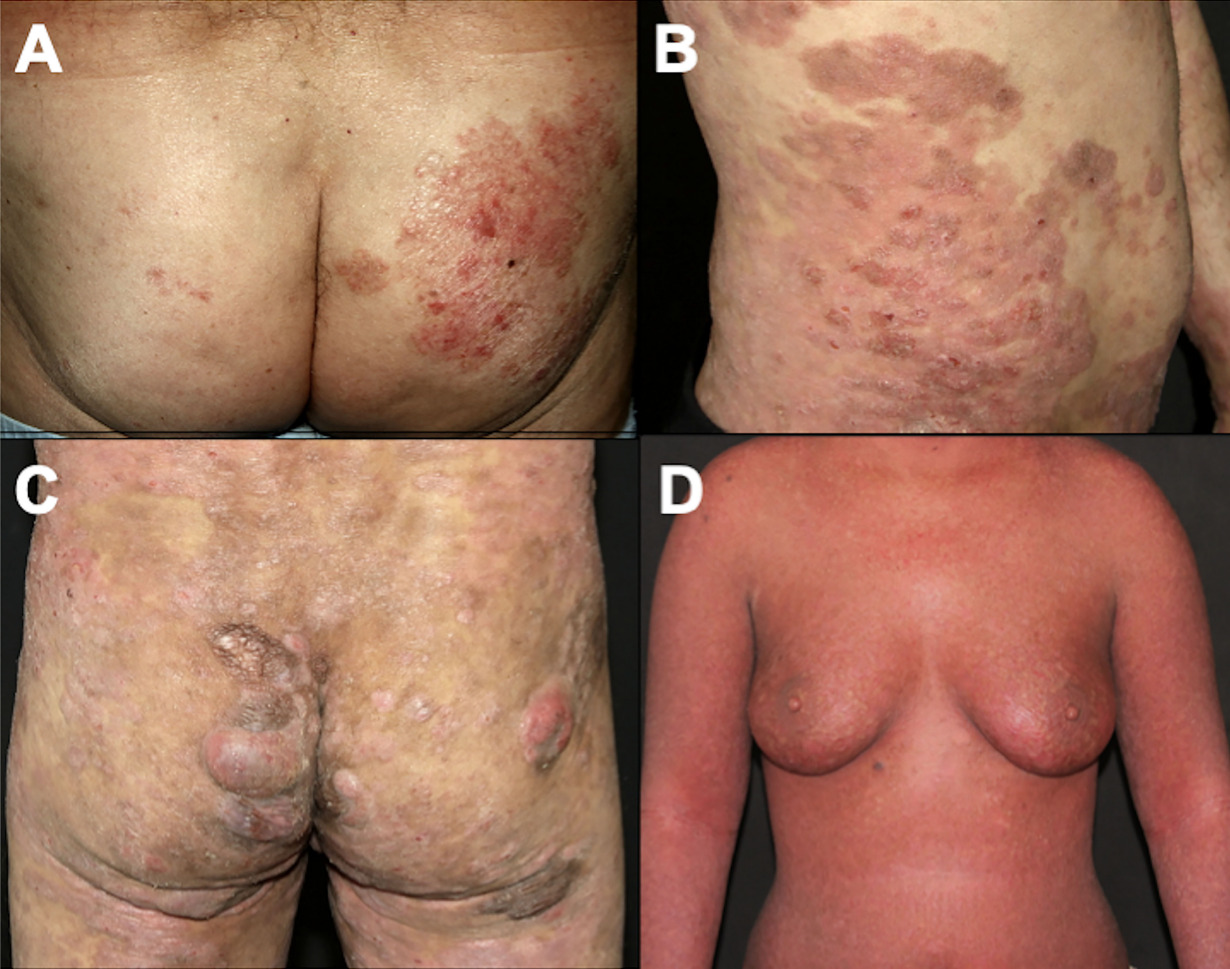
Clinical presentation of classic MF. Patches on the buttocks **(A)**; plaques on the abdomen **(B)**; patches, plaques, and tumors on the buttocks **(C)**; erythroderma **(D)**.

The classic type of MF, called the Alibert-Bazin type, is a progressive disease, with an indolent course. It is characterized by the development of patches, plaques, and tumors (**Figures 1A–C**) (3). Initially, there are non-infiltrated patches with erythema, scaling, and atrophy. These lesions may progress to erythematous infiltrated plaques, with well-defined borders and often with bizarre contours, with a foveolar, semi-annular, and serpiginous appearance, and eventually, tumors appear over the preexisting plaques or areas of healthy skin. It is common to observe a combination of lesions with patches, plaques, and tumors concomitantly. Not all MF patients progress from patches to plaques and tumors, but patches are always present (if a patient presents tumors without patches, other cutaneous lymphomas must be considered in the differential diagnoses). Thick infiltrated plaques and tumors may ulcerate, and erythroderma (involvement of more than 80% of the body surface area) may develop as the disease progresses (**Figure 1D**) (3, 56). A study with 1,502 patients with MF/SS showed that 71.4% had patches, 36.3% had plaques and 13.5% had tumors. Erythroderma was observed in 16.6% of cases (24).

The lesions initially appear on sun-protected areas, especially on the buttocks and breasts. The lower trunk, inguinal regions, axillae, and proximal areas of the upper and lower limbs are frequently involved. Lesions appear in variable numbers, and they spread gradually (3, 14).

Extracutaneous dissemination to lymph nodes, blood, or viscera is rare but has a significant negative impact on disease prognosis (57).

#### 4.1.2 Folliculotropic MF

**Figure 2:**
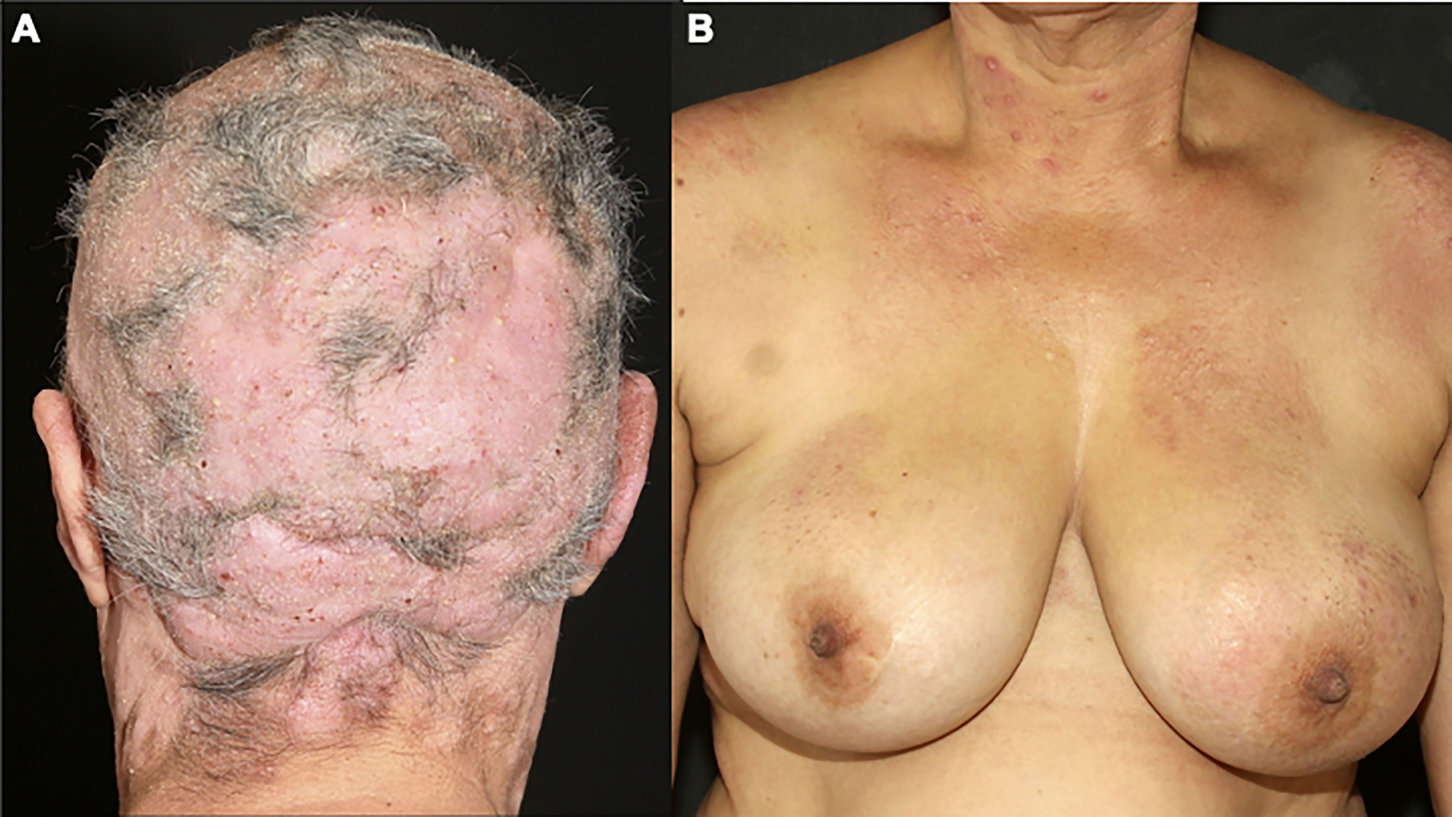
Folliculotropic MF. Advanced folliculotropic MF with infiltrated plaques on the scalp causing alopecia and milia **(A)**; early folliculotropic MF with patches and plaques with comedones and milia on the breasts, thorax, and neck **(B)**.

Folliculotropic MF is the most common variant, and it was considered a variant with a poorer prognosis, due to the presence of neoplastic infiltrate deeper in the dermis. However, recent studies divide the cases into advanced folliculotropic MF, with infiltrated plaques and tumors in the head and neck region with intense pruritus, cicatricial alopecia, and poorer prognosis (**Figure 2A**); and early folliculotropic MF, with patches and thin plaques with follicular accentuation, comedones, and milia in the trunk, milder pruritus, and a favorable prognosis (**Figure 2B**) (58, 59).

Not all patients with clinical features of folliculotropic MF have the description of folliculotropism on histopathology. This discrepancy occurs because sometimes the follicles are not represented in skin biopsy samples. On the other hand, there is the description of follicular involvement even in lesions that clinically do not suggest follicular involvement. The diagnosis of folliculotropic MF is based on the clinical picture in association with histopathology (60). Even in cases of childhood MF, including hypopigmented MF, folliculotropism can be observed, with no impact on the course of the disease (61). It is known that the hair follicle is a region of immune privilege, and the disruption of this barrier is observed in folliculotropic MF (62). It is still uncertain whether this breach of the immune privilege barrier also occurs in other MF variants in which folliculotropism can be observed on histopathology, and whether there is any impact on the prognosis. Furthermore, follicular accentuation may not always demonstrate infiltration of the follicular epithelium by neoplastic cells, but only follicular mucinosis, which can make confirmation of folliculotropic MF diagnosis difficult (63).

#### 4.1.3 Pagetoid reticulosis

**Figure 3:**
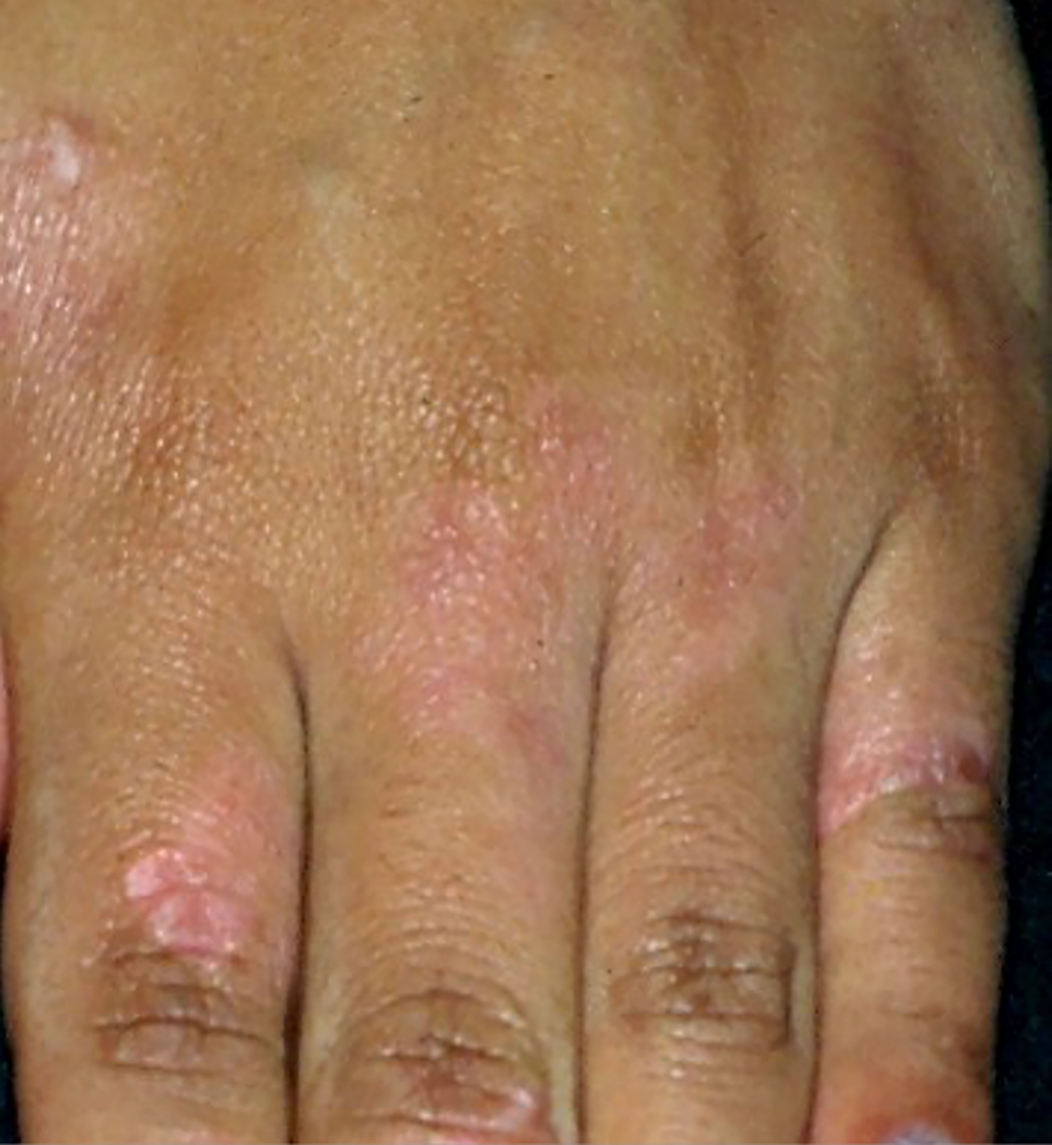
Pagetoid reticulosis. Psoriasiform plaques on the dorsum of the hand.

Pagetoid reticulosis is a rare and indolent MF variant. It is characterized by psoriasiform or hyperkeratotic lesions affecting the extremities (**Figure 3**). On histopathology, there is epidermal hyperplasia with a pagetoid proliferation of atypical T-lymphocytes with CD4+, CD8+, or CD4-CD8- phenotype (3, 64).

#### 4.1.4 Granulomatous slack skin

**Figure 4:**
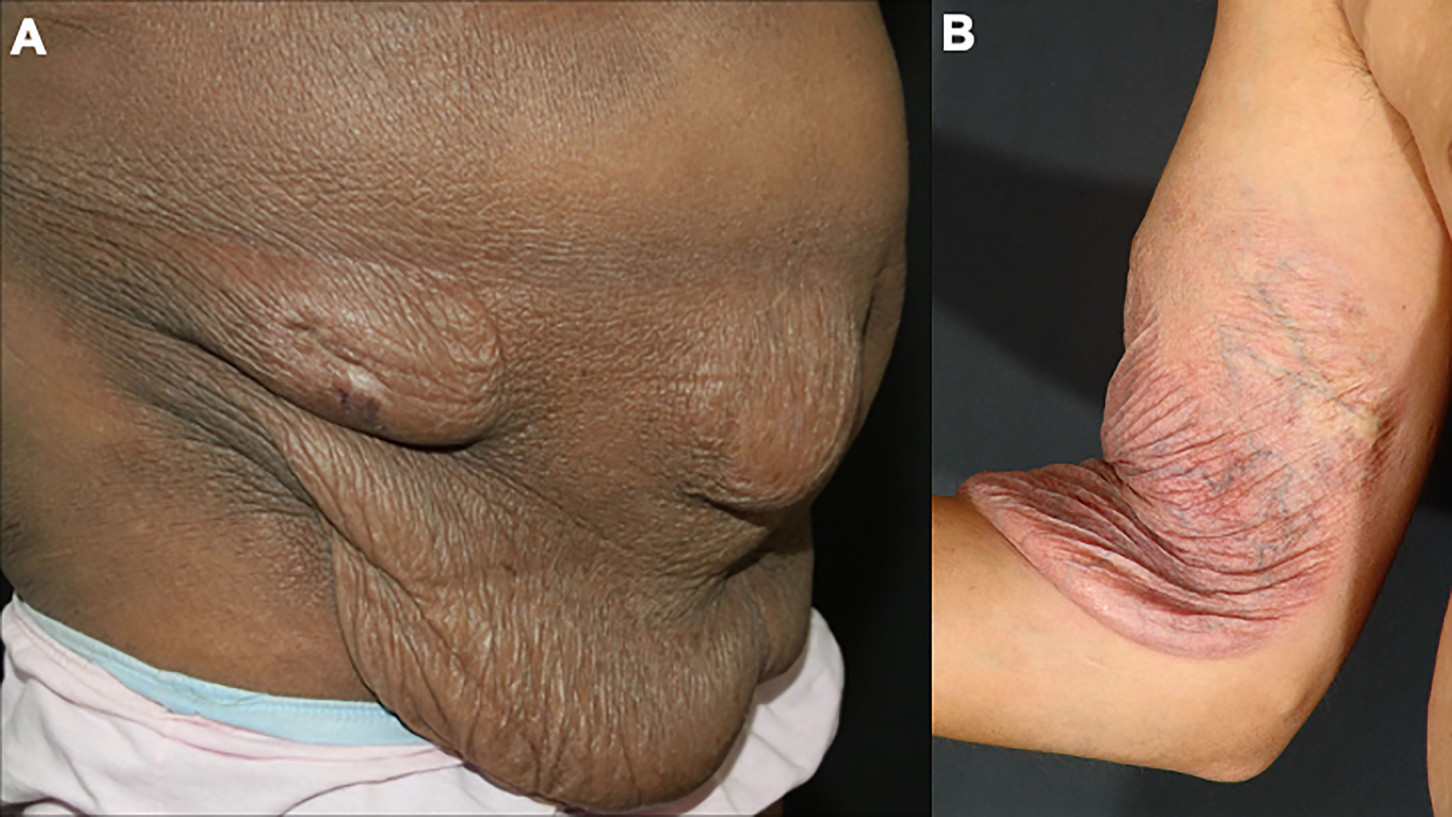
Granulomatous slack skin. Skin laxity on the abdomen **(A)** and the arm **(B)**.

Granulomatous slack skin is a rare and indolent MF variant, with peculiar clinical and histopathological characteristics. Initially, infiltrated papules and plaques appear on the skin folds, which may evolve with skin laxity (**Figure 4**). Histopathology shows granulomas, elastophagocytosis, and atypical lymphocytes infiltrating the skin. Patients with granulomatous slack skin have an increased risk of a second hematologic malignancy, especially anaplastic large T-cell lymphoma and Hodgkin’s lymphoma (65).

#### 4.1.5 Other clinicopathological subtypes

##### 4.1.5.1 Hypopigmented MF

**Figure 5:**
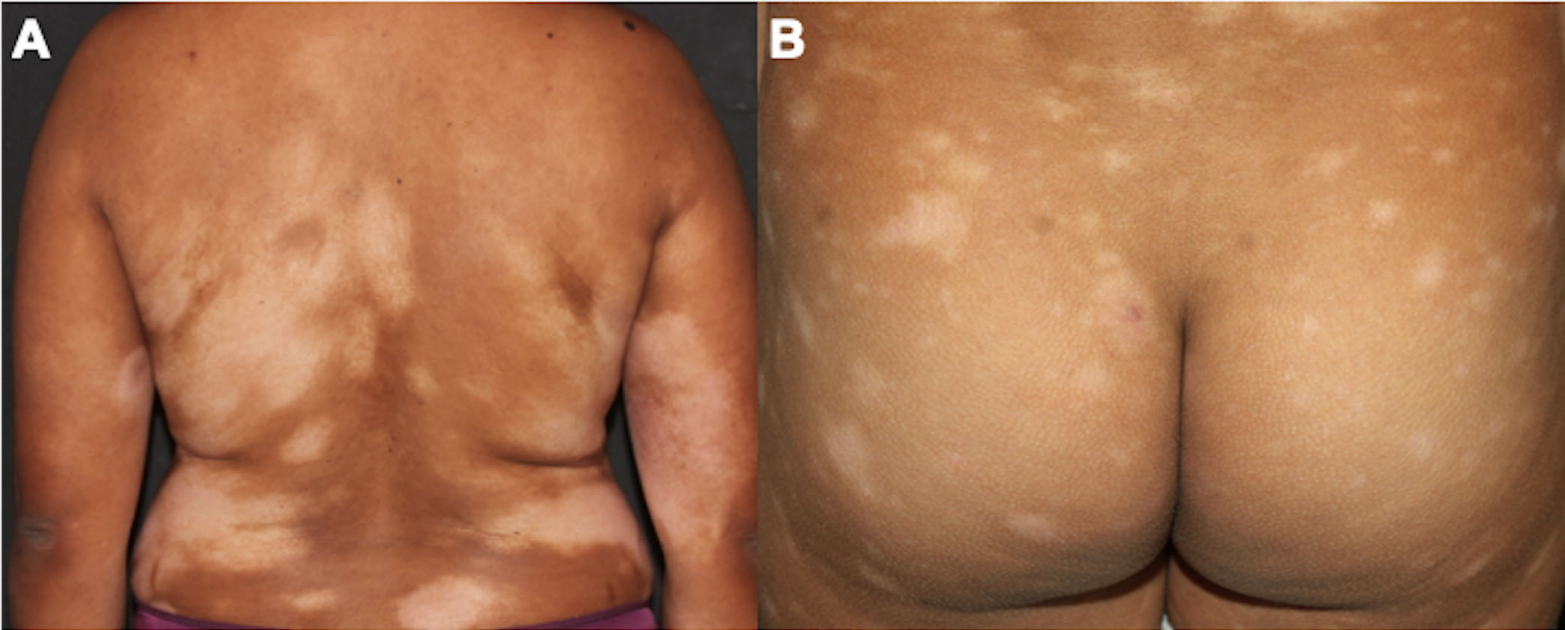
Hypopigmented MF. Multiple hypochromic patches on the trunk and arms **(A)**, and on the buttocks **(B)**.

The hypopigmented subtype of MF affects most frequently young patients with a median age at diagnosis of 32.2 years and with skin types IV-V (14, 15). An American study with 1,502 patients reported a diagnosis of hypopigmented MF in 3.4% of patients with MF (24). On the other hand, a Brazilian study reported a frequency of 19.6%, and studies with the pediatric population report hypopigmented MF in up to 50% of the total number of patients analyzed. Thus, geographical variations in the frequency of hypopigmented MF occur according to the ethnic characteristics of the populations (13, 15, 61, 66, 67). Hypopigmented MF is characterized by hypopigmented patches on the trunk, thighs, buttocks, and extremities (**Figure 5**) (15).

##### 4.1.5.2 Poikilodermatous MF

**Figure 6:**
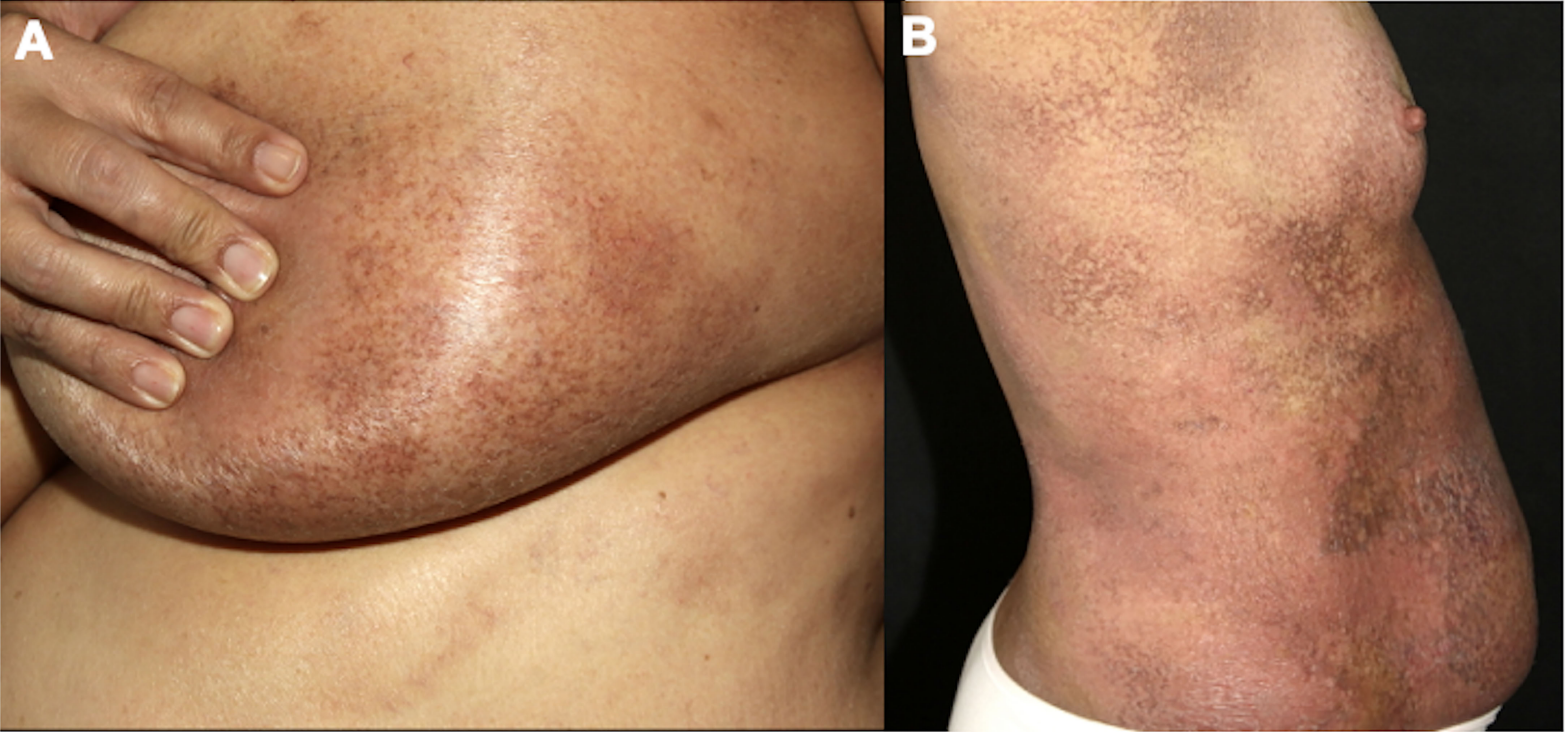
Poikilodermatous MF with localized **(A)** and generalized lesions **(B)**.

Poikilodermatous MF is characterized by hypopigmented, hyperpigmented, atrophic, and telangiectatic lesions, typically affecting flexural areas and the trunk. This type of MF accounts for 10% of cases and is more frequent in young patients (median age at diagnosis of 40 to 50 years) (51, 68). It can be subdivided into poikilodermatous MF with localized lesions and with generalized lesions (**Figure 6**). Patients with generalized poikilodermatous MF present erythroderma (more than 80% of the body surface area affected) and, despite the extensive skin area affected, the prognosis is excellent (51).

##### 4.1.5.3 Erythrodermic MF

**Figure 7:**
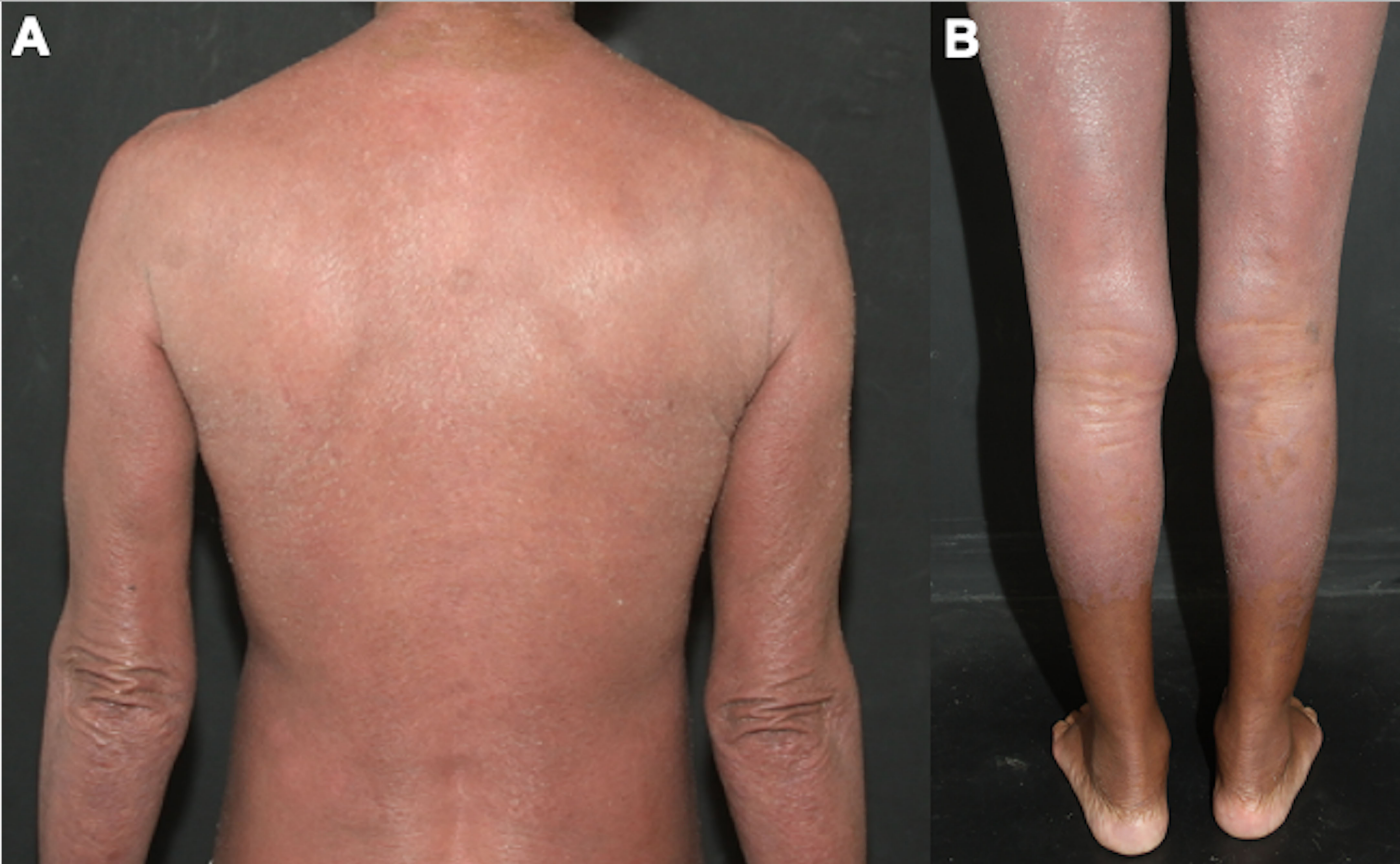
Erythrodermic MF. Difuse erythema and scaling affecting on the back and arms **(A)**, and on the lower limbs **(B)**.

The diagnosis and clinical management of erythrodermic MF are difficult. The patient presents with erythema and scaling on more than 80% of the body surface area (**Figure 7**), and the differential diagnoses include all causes of erythroderma, such as psoriasis, eczema, pityriasis rubra pilaris, drug eruption, and Sézary syndrome (69, 70). It is considered an aggressive MF variant, and peripheral blood assessment is critical to differentiate erythrodermic MF from SS (14, 71). It is discussed whether idiopathic cases of erythroderma may correspond to pre-malignant phases or even a type of MF in which it was not possible to identify neoplastic cells infiltrating the skin, due to the fewer epidermotropism of atypical lymphocytes observed in these erythrodermic cases (72).

##### 4.1.5.4 Other

Other rare clinicopathological subtypes of MF include granulomatous, hyperpigmented, ichthyosiform, syringotropic, papular, purpuric, interstitial, pustular, bullous, verrucous, and psoriasiform MF. These other subtypes are classified within the group of classic MF, and patients frequently present a mixed clinical presentation, together with the presence of patches, plaques, and tumors described by Alibert and Bazin (14, 49–51).

### 4.2 Sézary syndrome

**Figure 8:**
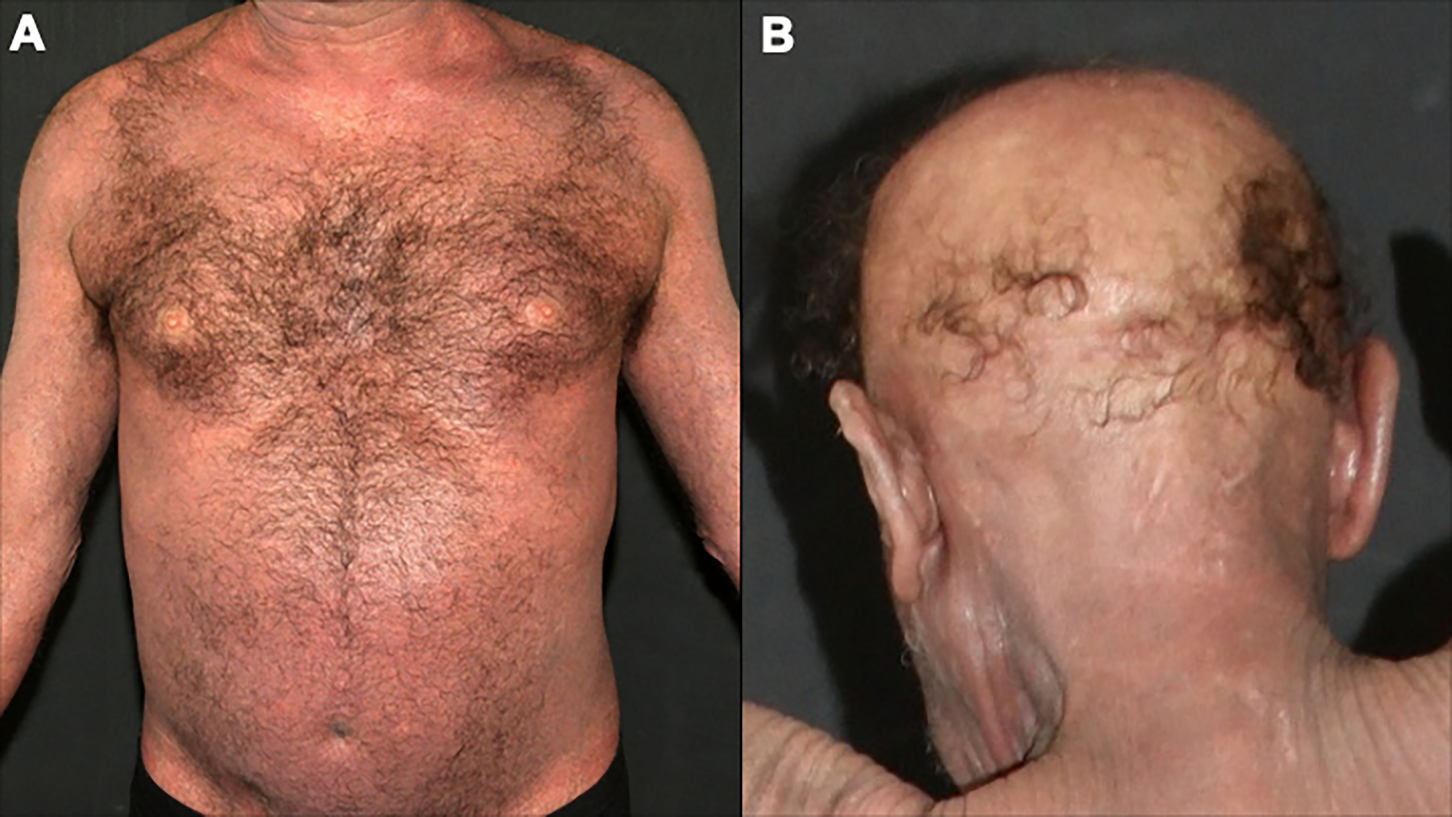
Sézary syndrome. Diffuse erythema and scaling **(A)** and non-scarring alopecia **(B)**.

**Figure 9:**
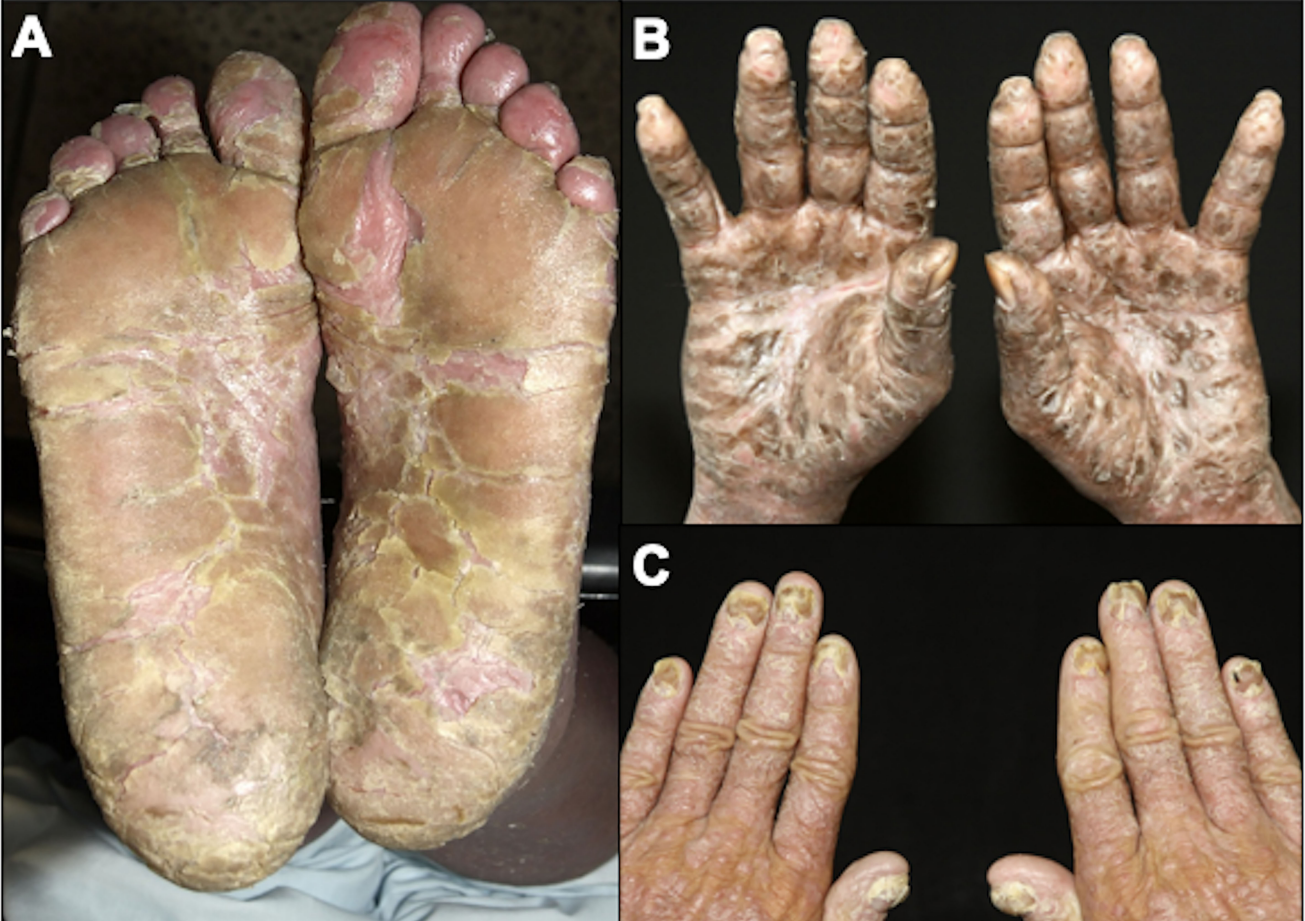
Sézary syndrome. Plantar **(A)** and palmar **(B)** keratoderma, onychodystrophy **(C)**.

SS is a leukemic variant of CTCL and occurs almost exclusively in adults. It presents with erythroderma with a diffusely infiltrated aspect of the skin, often associated with non-scarring alopecia, palmoplantar keratoderma, nail dystrophies, generalized lymph nodes enlargement, and intense itching (**Figures 8**, **9**). Circulating neoplastic T-cells are detected by searching for Sézary cells (large lymphocytes with a cerebriform nucleus) in the blood smear and by immunophenotyping of lymphocytes by flow cytometry. Search for T-cell receptor (TCR) gene rearrangement detects monoclonal populations in peripheral blood, skin, and lymph nodes. For the diagnosis of SS, the International Society for Cutaneous Lymphomas (ISCL) propose that the presence of a monoclonal population in the skin and blood (same clone) and one of the phenotypic alterations (CD4/CD8 ≥ 10 and/or CD4+CD7- ≥ 40% and/or CD4+CD26- ≥ 30%) or detection of more than 1,000 Sézary cells/µL are mandatory (**Table 1**) (3, 10, 73). According to the most recent World Health Organization (WHO) classification, SS diagnosis also requires erythroderma, generalized lymphadenopathy, and clonally related malignant T-cells in the skin, peripheral blood, and lymph nodes (74).

**Table 1:** Sézary syndrome diagnostic criteria.

Sézary cells in peripheral blood smear	≥ 1,000 Sézary cells/µL
Immunophenotyping of lymphocytes in peripheral blood	CD4/CD8 ≥ 10
	CD4+CD7- ≥ 40%
	CD4+CD26- ≥ 30%
T-cell receptor gene rearrangement	Monoclonal population on the skin and peripheral blood (same clone)

*For the diagnosis of SS, detection of a monoclonal population of T-cells on the skin and the blood is mandatory together with at least one morphologic or immunophenotypic alteration.

## 5 Skin biopsy

**Figure 10:**
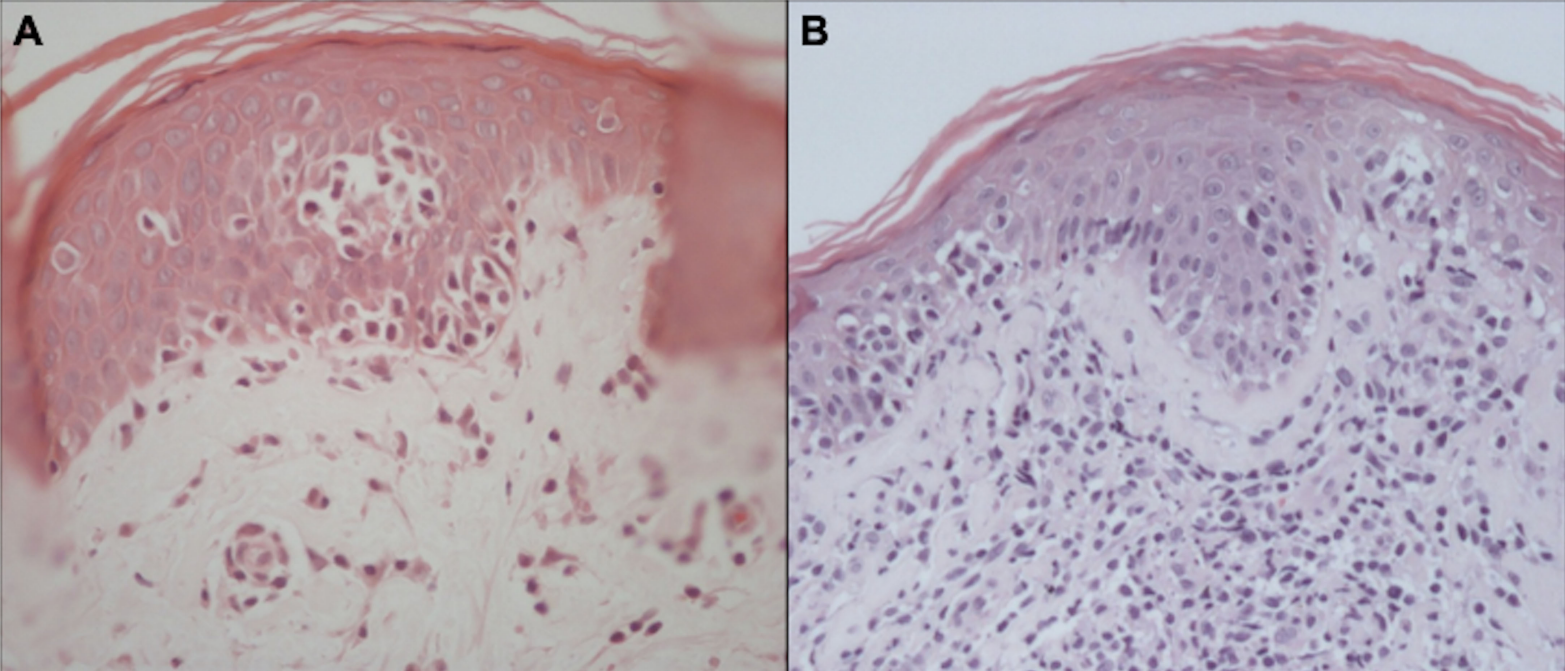
Histopathology of MF. Epidermotropism of atypical lymphocytes with Pautrier microabscess and scant dermal lymphocytic infiltrate **(A)**. Epidermotropism, Pautrier microabscesses, and a more prominent dermal lymphocytic infiltrate **(B)**.

The confirmation of MF/SS diagnosis is difficult. There are no “gold standard” tests, and diagnosis is based on clinical, histopathological, and molecular findings. On histopathology, the diagnosis of the neoplasm is usually suggested by experienced pathologists by the cytomorphological characteristics and the disposition of the architectural arrangement of the infiltrate. Currently, for the classification of lymphomas, it is essential to carry out an immunohistochemical study, whose panel of antibodies is rationalized according to the histological findings (75). It is recommended to biopsy different lesions simultaneously to increase the accuracy of histopathology (14). In MF, the cytological aspects and the architectural pattern of the cellular infiltrate are correlated with the clinical stage of the disease. Epidermotropism of atypical lymphocytes is the most striking feature. Atypical lymphocytes have a large and hyperchromatic nucleus, sometimes with cerebriform contours (76). In the early patch stages and in the erythrodermic phase, the number of atypical lymphocytes infiltrating the epidermis is scarce, and it increases in plaques and tumors (77). However, sometimes epidermotropism may be absent even in plaques and tumors, making the diagnosis if MF more difficult. Other typical pathological findings are Pautrier’s microabscesses (grouping of at least 4 lymphocytes around a Langerhans cell in the epidermis) (**Figure 10**) (78, 79). The alignment of lymphocytes in the basal layer may also be observed in MF (80). The absence of spongiosis favors the diagnosis of MF (78); however, it has low specificity (60%) (81).

Syringotropic, granulomatous, and interstitial MF are histological MF variants rarely described in the literature. Syringotropic MF is more commonly seen in palmoplantar lesions (82). Granulomatous MF should be distinguished from granulomatous slack skin based on the clinical picture (in granulomatous MF there are no areas of lax skin) and histopathology (elastolysis and elastophagocytosis are seen in granulomatous slack skin) (65).

Large cell transformation (LCT) is a histopathological finding characterized by the presence of large cells (cells four times larger than normal lymphocytes) in 25% or more of the dermal infiltrate, or when these large cells are grouped in nodules (the definition of nodules of large cells is vague, most pathologists do not use it, and these nodules can be focal and hard to identify in skin or lymph node biopsies) (83). It can occur in tumor lesions and, less frequently, in plaque and erythrodermic MF lesions. These cells may or may not express CD30, and it is essential to differentiate MF with LCT from CD30-positive anaplastic large T-cell lymphoma (73). A higher frequency of CD56 expression is reported in MF with LCT compared to non-transformed MF, however, the clinical significance of this finding is not clear (84). The detection of LCT confers a poorer prognosis (57).

### 5.1 Immunohistochemistry

**Figure 11:**
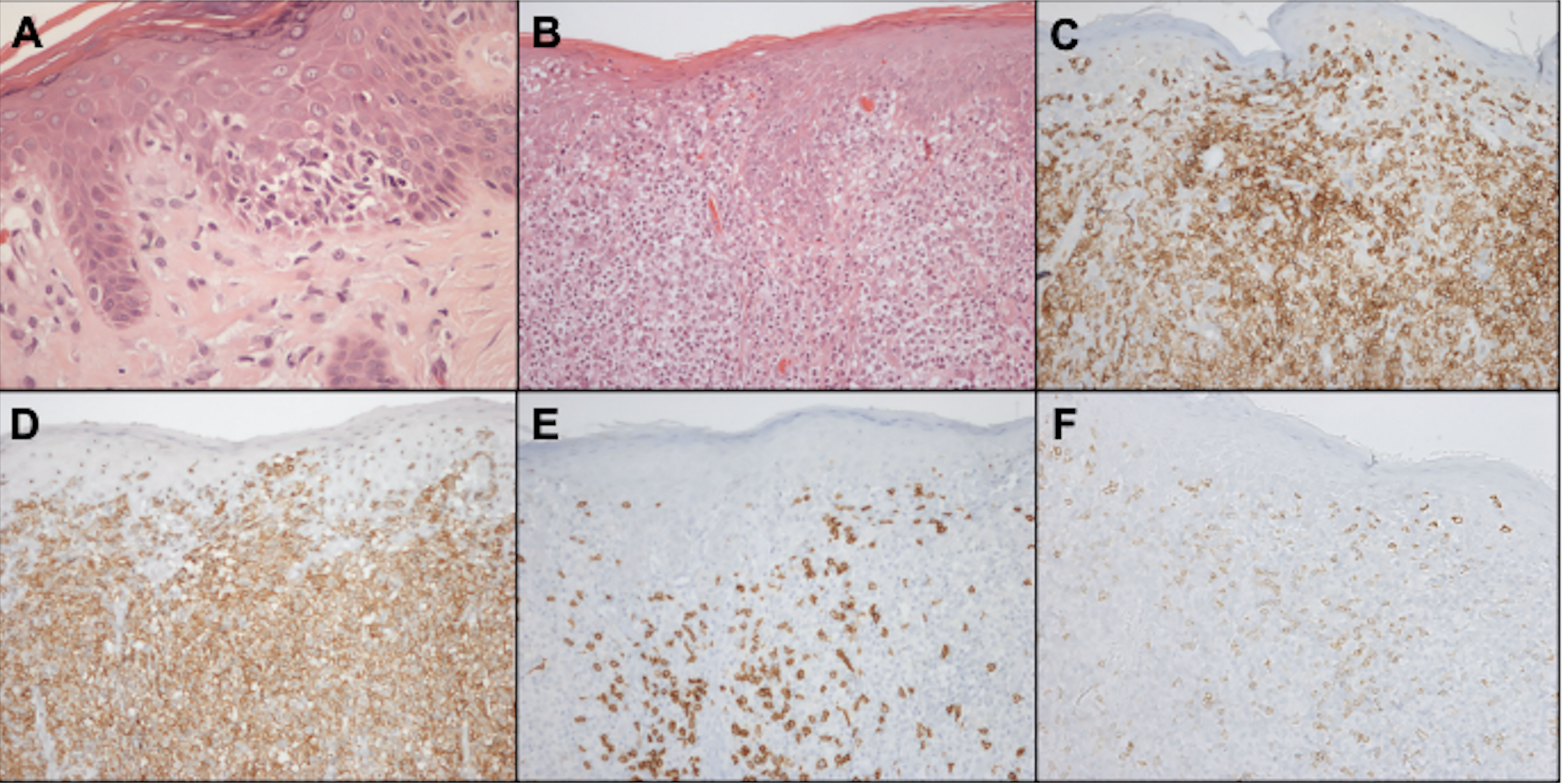
Histopathology and immunohistochemistry of the skin. Epidermotropism of atypical lymphocytes, hematoxylin-eosin staining at 400x magnification **(A)**. Exuberant atypical infiltrate affecting the epidermis and dermis, hematoxylin-eosin at 200x magnification **(B)**. CD3 positivity, demonstrating that epidermal and dermal cells correspond to T-lymphocytes, 200x magnification **(C)**. CD4 positivity, 200x magnification **(D)**. Expression of CD8 on small reactive cells, 200x magnification **(E)**. Loss of CD7 expression in CD4-positive neoplastic T cells, 200x magnification **(F)**.

Malignant cells are CD4-positive T-cells with a memory phenotype (CD3+CD4+CD45RO+CD8–) and negativity for CD7 antigen expression in 70% of cases (**Figure 11**). Rarely, neoplastic cells express a CD3+CD4-CD8+ phenotype, especially in hypopigmented MF, with the same clinical behavior and prognosis as cases with CD4+CD8- cells. Losses of CD2 and CD5 are observed less frequently (56, 85, 86).

CD30 is a molecule of the tumor necrosis factor family and is expressed on the cell surface of activated B and T-lymphocytes. Search for CD30 expression in MF is important, especially in advanced-stage cases. The brentuximab-vedotin, a drug-antibody conjugate in which the monomethyl auristatin E (MMAE) molecule, an antimicrotubule agent, is linked to an anti-CD30 monoclonal antibody, was approved for the treatment of CD30+ MF refractory to prior systemic treatment. This drug has shown good responses in cases of MF/SS that express CD30 in at least 10% of neoplastic cells in a phase 3 clinical trial (87). Studies show CD30 expression in most patients (88), but its expression is low, with an average expression in 4% of neoplastic cells (89).

Despite its relationship with cell proliferation, the Ki-67 expression was not consistently associated with poorer prognosis (57), but a higher expression is observed in cases of large cell transformation (89).

There are reports of phenotypic variability of neoplastic cells in the same patient. These variations have uncertain clinical significance, but it is important to emphasize the need to perform biopsies in different topographies and repeatedly in cases where the clinical suspicion of MF/SS is not supported by the histopathological examination (90).

In SS, histopathology is similar to MF. However, in erythrodermic forms of cutaneous lymphomas (erythrodermic MF and SS) epidermotropism is less evident, and in up to a third of SS cases the histopathology may be nonspecific (10).

### 5.2 T-cell clonality in the skin

The search for TCR gene rearrangement, demonstrating monoclonal T lymphocyte proliferation in the skin, lymph nodes, and/or peripheral blood, may contribute to the diagnosis of T-cell lymphomas. It is done by polymerase chain reaction (PCR), Southern blot, or more recently by next-generation sequencing (NGS) (91–93).

T-cell monoclonality is observed in 50% of patch lesions, 73% of plaque lesions, 83 to 100% of tumors, and 77.5% of patients with SS, and the detection of the same clone in different skin lesions favors the diagnosis of CTCL. On the other hand, 25 to 65% of inflammatory dermatoses may exhibit oligoclones in the skin. Therefore, the presence of a monoclonal population should be analyzed with caution, especially in cases where histopathology does not confirm the diagnosis of MF/SS (10, 78, 94).

## 6 Staging

Early-stage diseases (stages IA to IIA) account for 78% to 93% of MF/SS cases; advanced-stage (≥IIB) corresponds to 7% to 22% (21, 95, 96).

### 6.1 Skin (T)

For MF and SS, the modified TNM system (tumor, lymph nodes, and metastasis) of Bunn and Lamberg (1979) was adopted, considering T as cutaneous lesions, N as lymph nodes, M as visceral lesions, and adding B as peripheral blood (**Tables 2**, **3**) (73, 97, 98).

**Table 2:** Revised TMNB classification for MF and SS.

Skin (T)	
T1	Limited patches/plaques (covering <10% of the total skin surface)
T1a	Patches only
T1b	Plaques/papules ± patches
T2	Generalized patches/plaques (covering ≥10% of the total skin surface)
T2a	Patches only
T2b	Plaques/papules ± patches
T3	Tumor(s) ≥ 1cm diameter
T4	Confluence of erythema covering ≥80% of the body surface area
Lymph node (N)	
N0	No clinically abnormal peripheral lymph nodes
N1	Clinically abnormal peripheral lymph nodes; dermatopathic lymphadenopathy or histopathological involvement by isolated atypical lymphocytes, without alteration of the lymph node architecture
N1a	Clone negative or equivocal
N1b	Clone positive and identical to skin
N2	Clinically abnormal peripheral lymph nodes; histopathological involvement by aggregates of atypical lymphocytes, without alteration of the lymph node architecture
N2a	Clone negative or equivocal
N2b	Clone positive and identical to skin
N3	Clinically abnormal peripheral lymph nodes; frank histopathological involvement and partial/complete effacement of the lymph node architecture
N3a	Clone negative or equivocal
N3b	Clone positive and identical to skin
Nx	Clinically abnormal peripheral lymph nodes; without histopathological confirmation
Viscera (M)	
M0	Without visceral involvement
M1	With visceral involvement
M1a	Bone marrow involvement
M1b	Non-bone marrow visceral involvement
Blood (B)	
B0	Absence of significant blood involvement (< 250/µL of CD4+CD26- or CD4+CD7- cells)
B0a	Clone negative or equivocal
B0b	Clone positive and identical to skin
B1	Low tumor burden in the blood, but do not fulfill B2 criteria
B1a	Clone negative or equivocal
B1b	Clone positive and identical to skin
B2	High tumor load in the blood (CD4/CD8 ≥ 10; CD4+CD7- ≥ 40%; CD4+CD26- ≥ 30%; ≥ 1000/µL of CD4+CD26- or CD4+CD7- cells or other aberrant population of lymphocytes identified by flow cytometry)
B2a	Clone negative or equivocal
B2b	Clone positive and identical to skin
Bx	Unable to quantify blood involvement

**Table 3:** Clinical staging system for MF and SS.

Clinical staging	TNMB classification			
IA	T1	N0	M0	B0 or B1
IB	T2	N0	M0	B0 or B1
IIA	T1 or T2	N1 or N2	M0	B0 or B1
IIB	T3	N0 to N2	M0	B0 or B1
IIIA	T4	N0 to N2	M0	B0
IIIB	T4	N0 to N2	M0	B1
IVA1	T1 to T4	N0 to N2	M0	B2
IVA2	T1 to T4	N3	M0	B0 to B2
IVB	T1 to T4	N0 to N3	M1	B0 to B2

The prognosis of patients with MF is directly associated with the clinical stage. Progressively poorer survival curves are observed depending on the extent of cutaneous involvement, presence of tumors and erythroderma, lymph node, visceral, and peripheral blood involvement (73). A study with 489 MF patients showed that T1 patients had survival similar to the general population. However, T2, T3, and T4 patients had significantly lower survival rates. T2 patients with plaque stage (T2b) had poorer survival than T2 patients with patches only (T2a) (99). The presence of a tumor greater than or equal to 1 cm in diameter is sufficient to define the T3 stage. In the presence of more than one T category, the highest one must be included, and in cases of erythroderma (T4) associated with tumor lesions (T3), both forms must be recorded [T4 (3)] (73).

### 6.2 Lymph nodes (N)

Around 80% of patients have no clinical suspicion of lymph node involvement at the diagnosis (N0) (24, 95).

A biopsy of lymph nodes greater than or equal to 1.5 cm in diameter or of any palpable lymph node, regardless of size, that is hardened, irregular, fixed, or forming a conglomerate of lymph nodes is recommended. Lymph node enlargement can be confirmed by ultrasound, computed tomography (CT), positron emission tomography (PET), or magnetic resonance imaging, before the biopsy is performed. Excisional biopsy is preferred, except in cases with a high risk of infection, especially erythrodermic patients, when core needle biopsy may be indicated. In the presence of multiple altered lymph nodes, the order of preference of biopsy is cervical, axillary, and inguinal lymph nodes, as cervical lymph nodes are more likely to demonstrate lymphomatous involvement (73, 100, 101).

Erythrodermic patients, such as patients with SS, regardless of the erythroderma etiology, frequently have enlarged lymph nodes (69, 70). This occurs due to intense skin inflammation in erythrodermic patients. Even in SS cases, almost 20% have no lymph node involvement by the disease confirmed by histopathology, and dermatopathic lymphadenitis is detected (10).

#### 6.2.1 Histopathological examination of the lymph node

**Figure 12:**
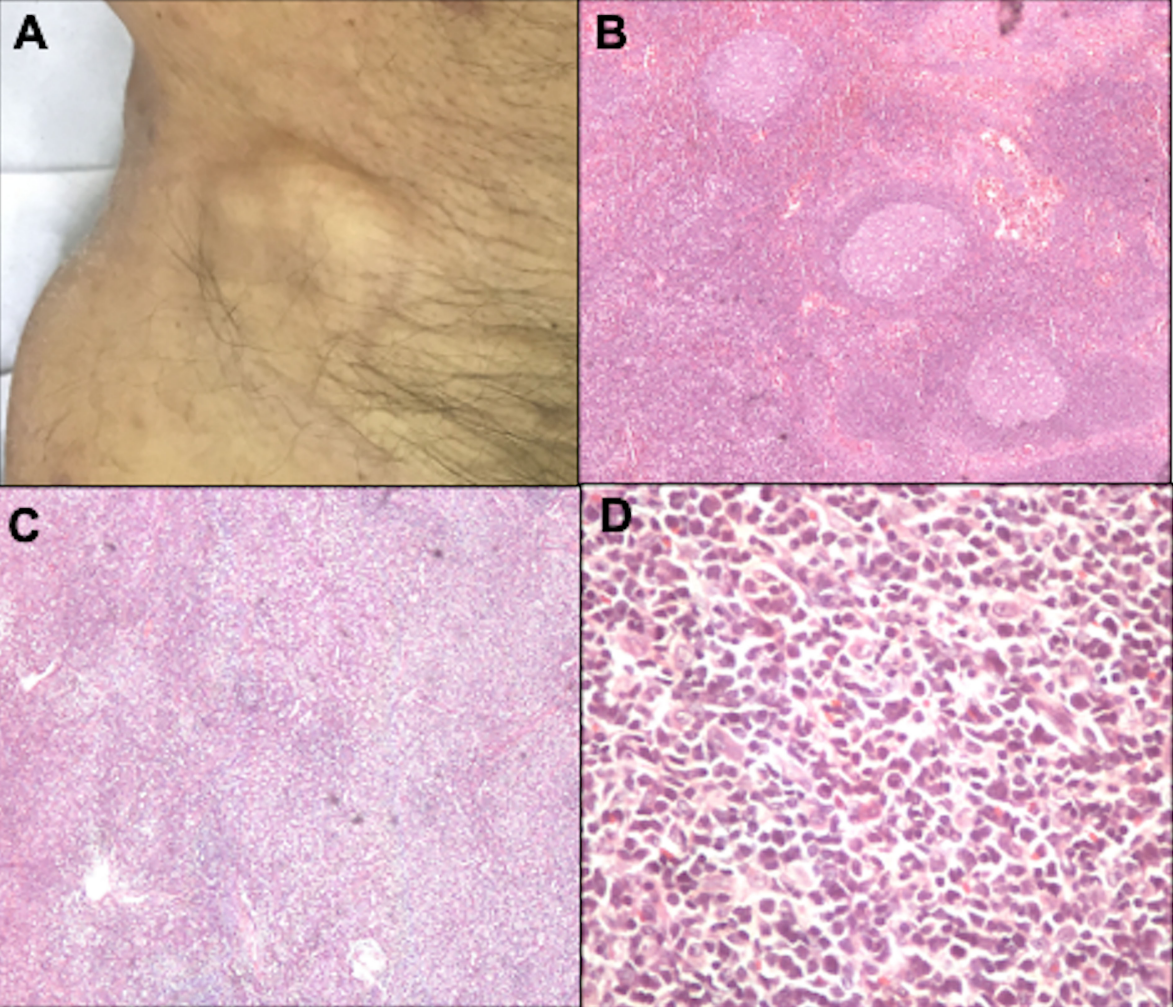
Lymph node evaluation. Inguinal lymph node enlargement **(A)**. Dermatopathic lymphadenopathy with the presence of germinal centers, hematoxylin-eosin at 20x magnification **(B)**. Lymph node involvement by lymphoma, with architectural alteration, hematoxylin-eosin at 20x magnification **(C)**. Pleomorphic lymphoid cells, hematoxylin-eosin at 200x magnification **(D)**.

Dermatopathic lymphadenopathy (N1) is characterized by paracortical layer hyperplasia, usually secondary to chronic cutaneous inflammation (102). The N2 stage corresponds to partial lymph node involvement; and N3, to complete involvement with an architectural alteration (**Figure 12**). In the literature, nodal involvement (N2 or N3) is observed in 30 to 50% of biopsied cases (24, 103).

#### 6.2.2 Search for T clonality in the lymph node

Studies show lymph node T-cell monoclonality in 77.8% to 81.5% of patients with SS (10, 104). Even in patients with MF and biopsy with dermatopathic lymphadenopathy (N1), the presence of a monoclonal population can be detected (stage N1b), and studies suggest that the presence of this clone confers a poorer prognosis (104). However, more studies are needed to assess whether systemic treatments change the course of these patients with early MF and N1b stage, who would usually be treated with skin-directed therapies.

### 6.3 Viscera (M)

Regarding the M stage, splenomegaly detected on physical examination or imaging studies is considered a visceral disease regardless of histological confirmation. The suspicion of involvement of other organs, through clinical, laboratory, or imaging evaluation, must be confirmed by a biopsy (73).

Less than 1% of MF/SS cases have visceral involvement at the diagnosis, and its presence is associated with a poorer prognosis, with a median overall survival of 33.3 months (57, 95).

It is recommended that imaging tests be performed for the staging of all patients with MF/SS. For early-stage disease, chest radiography and abdominal and lymph nodes ultrasonography may be performed. In advanced cases, CT of the chest, abdomen, and pelvis, PET scan, or MRI are indicated depending on the organ to be evaluated (73). Studies show that the PET scan is more accurate in diagnosing nodal involvement in patients with MF/SS, but this test is not available in all medical centers, and its high cost makes it difficult to perform routinely (103).

#### 6.3.1 Visceral biopsies

An autopsy study with 45 MF patients observed lymph node and visceral involvement in 24 cases (53.3%), particularly in patients with advanced disease (stage IIB-IVB) (105, 106). Another study with autopsies of MF/SS patients observed that the most affected extracutaneous sites are: lymph nodes (60%), spleen (50%), lungs (43%), liver (41%), bones (27%), kidneys (27%), tongue and mucous membranes (19%), heart (17%), pancreas (17%), thyroid (14%) (107). Currently, autopsy studies are scarce, but these older studies show that visceral involvement may be more frequent than what is detected. However, the impact of visceral infiltration not detected clinically or by imaging methods (only at autopsy) on outcome and prognosis is unknown.

#### 6.3.2 Myelogram and bone marrow biopsy

Bone marrow biopsy is indicated in patients with significant peripheral blood involvement (stage B2) or when there are hematological abnormalities that cannot be explained by other causes. Bone marrow involvement in MF/SS is rare and is considered visceral involvement (M1) (24, 73). A myelogram is rarely indicated (24). The role of bone marrow biopsy in MF/SS is not well established, and studies on the impact of bone marrow involvement on disease prognosis are conflicting (108, 109). A study with SS patients showed that 31.6% of cases had bone marrow involvement, but there was no negative impact on survival in cases with bone marrow infiltration (10). A study on autopsies of patients with MF/SS showed bone marrow involvement in 25% of patients. On the other hand, a study with 50 patients with different stages of cutaneous T-cell lymphoma did not observe bone marrow involvement in any case (105). Benign lymphoid nodules can be seen in the bone marrow of healthy people, and the definition of tumor infiltration depends on the criteria used (10, 105). Thus, it is necessary to standardize the histopathological analysis to assess the presence of malignant T-cells in the bone marrow. Studies evaluating clonality in the bone marrow are scarce, but one study demonstrated T-lymphocyte clones in 53.6% (15/28) of patients with SS (10).

### 6.4 Blood (B)

Hematologic involvement is assessed by peripheral blood smear (search for Sézary cells), immunophenotyping of lymphocytes by flow cytometry, and search for T-cell clonality. Hematologic involvement is stratified into B0 (no blood involvement, ≤ 5% Sézary cells or < 250/µL of CD4+CD26- or CD4+CD7- cells), B1 (low blood tumor burden, with > 5% Sézary cells or ≥ 250/µL of CD4+CD26- or CD4+CD7- cells, but not meeting the criteria for B2), and B2 (high tumor burden in the blood, with ≥ 1,000 Sézary cells/μL, an increase in CD4+ cells with a CD4/CD8 ratio ≥ 10, CD4+/CD7- ≥ 40%, CD4+/CD26- ≥ 30%, ≥ 1000/µL of CD4+CD26- or CD4+CD7- cells, or other aberrant population of lymphocytes identified by flow cytometry). The B stages may be further subdivided into B0a, B1a, B2a (negative blood clonality) or B0b, B1b, B2b (positive blood clonality) (73, 98, 100, 110).

#### 6.4.1 Search for Sézary cells in peripheral blood smear

The morphological evaluation of lymphocytes on a peripheral blood smear by experienced hematopathologists is an easy and convenient tool to differentiate patients with erythrodermic cutaneous lymphomas and erythroderma caused by inflammatory skin conditions. However, with the advancement of flow cytometry techniques, the evaluation of lymphocyte morphology by peripheral blood smear is less frequently performed. This stems from technical difficulties, such as high time consumption and imprecision of manual search for Sézary cells, especially when these cells are small. In addition, phenotypic alterations occur earlier than morphological alterations (111).

#### 6.4.2 Immunophenotyping of lymphocytes by flow cytometry

Although flow cytometry has become the standard tool for the evaluation of peripheral blood, there is no consensus and standardization regarding the markers used and the way to report this test. Ideally, it would be necessary to include CD3 and CD19 in the studied panel, to differentiate T and B-lymphocytes, respectively. Subsequently, T-lymphocytes should be better characterized, with the evaluation of CD4 and CD8, as well as CD7 and CD26, which are lost in neoplastic cells in MF/SS. Other T-lymphocyte markers, such as CD3, CD2, and CD5, are rarely lost, however, a diminished intensity or partial loss may be observed in some cases (112). CD52, CD25, CD30, and CCR4 are other markers that can aid in the therapeutic decision, as these molecules are targets of specific monoclonal antibodies (alemtuzumab, denileukin diftitox, brentuximab-vedotin, mogamulizumab, respectively) (111, 113).

Preliminary data from the PROCLIPI study show changes in the peripheral blood of patients with early MF with patches and plaques, fulfilling criteria for B1, in up to 5.9% of patients (114). The clinical significance of these changes in early MF is uncertain, and it is unknown whether they would be cases of true hematological involvement of MF. In erythrodermic patients with peripheral blood involvement compatible with stage B1, there is a greater risk of disease progression (115).

In SS, in addition to the increased CD4-positive T-cells, CD8 cytotoxic T-cells are reduced. Also, functional alterations of CD8 and NK cells are described, leading to a state of immunosuppression by compromising the innate immune response (116, 117).

#### 6.4.3 T-cell clonality in blood

Preliminary data from the PROCLIPI study show that the frequency ofmonoclonal T-cell population in the blood matching the skin clone of patients with MF increases with higher stages (114). In patients with SS, clonality in the blood is positive in 86.7% to 100% of patients (10, 118). However, the presence of T-cell clones in the blood is not always a sign of malignancy, since clones may be observed in healthy elderly individuals and patients with benign dermatoses, and the significance of these circulating clones is unknown (119, 120).

### 6.5 Other laboratory tests

Lactate dehydrogenase (LDH) is an enzyme that catalyzes the conversion of lactate to pyruvate and vice versa in the process of glycolysis. Its activity is high in tumor cells of several malignancies (121). When increased at the diagnosis, a poorer prognosis is observed in MF/SS (57). An increase in LDH is observed in 25% of MF patients (10, 13, 122, 123).

Beta-2 microglobulin is part of the major histocompatibility complex class 1 and is present in all nucleated cells. Increased levels are associated with poorer prognosis in several hematologic malignancies, but the mechanism of association between beta-2 microglobulin and prognosis is unknown. Studies suggest that this molecule is involved in the survival and proliferation of malignant cells, as well as their ability to metastasize (124). In MF, studies demonstrate the association between its levels and disease progression, and higher levels of beta-2 microglobulin are detected in patients with late-stage MF compared to early MF, and in SS compared to MF.

Low albumin levels are associated with poorer prognosis in nodal lymphomas, and it is used as a predictor of response to first-line chemotherapy for peripheral T-cell lymphomas (125). In erythrodermic patients, the intense protein loss due to the universal skin alteration may cause hypoalbuminemia (126, 127). Hypoalbuminemia is more frequent in patients with advanced MF (20.9%) compared to early MF (5.4%), and in patients with SS (30.8%) compared to patients with MF (11.7%) (13). However, in multivariate analyses hypoalbuminemia was not associated with poorer prognosis in cutaneous lymphomas (13, 24, 57).

## 7 Prognosis

Lymphomas that present primarily in the skin, without evidence of extracutaneous disease at the diagnosis, often have a more indolent clinical behavior and a better prognosis compared with systemic lymphomas of a similar histological subtype (3).

In MF, patients with skin-limited disease have a good response to topical treatments and the overall survival is similar to the healthy population. Only 2% of the patients with localized lesions (involvement of less than 10% of the body surface area) die after 32 years of follow-up and only 9% show progression to plaques and tumors with lymph node, blood, and visceral dissemination (11). The 5-year overall survival is 91 to 97% for patients with non-infiltrating patch lesions or localized plaques (<10% of the skin surface), 81 to 85% for patients with generalized lesions (≥10% of the skin surface), 44% for patients with tumors, and 20 to 30% for those with lymph node disease. Sepsis, especially caused by *Staphylococcus aureus*, represents one of the most frequent causes of death in advanced cases (24). Transformed MF has a poorer prognosis, with a 5-year overall survival of 38.5% (57). SS is an aggressive lymphoma, with five-year survival rates ranging from 40 to 50% (3, 10, 57, 128, 129).

The CLIC study proposed a prognostic stratification index based on age (≥ 60 years), increase in LDH, large cell transformation, and stage IV at diagnosis (57). However, even this prognostic index proposed by this international multicentricstudy was not applicable in all series (130). Other factors reported to be associated with poorer prognosis are advanced age (20, 24, 131), male gender (24), folliculotropism in histopathology in patients with infiltrated plaques and tumors in the head and neck region (24, 58, 132–134). However, the results of studies on prognostic factors in MF/SS are conflicting. Such prognostic studies are mostly retrospective observational studies. The PROCLIPI study is an international multicenter prospective study that was derived from the CLIC study, which was retrospective. It aims to develop a prognostic index to achieve better stratification and management of patients (135).

## 8 Treatment

Watch and wait may be indicated in cases of stage IA MF that are at low risk of progression and with survival similar to the general population for their gender and age (136).

For early-stage patients with no extracutaneous involvement, skin-directed therapies are used with good response rates. For advanced-stage disease with multiple tumors, erythroderma, or extracutaneous disease, or early-stage with multiple infiltrated plaques refractory to topical therapy, systemic treatments are indicated (137).

Skin-directed therapies include topical steroids, nitrogen mustard, bexarotene, phototherapy with narrow-band UVB and PUVA, and radiation therapy (localized and total skin electron beam irradiation) (113, 138, 139). Topical tacrolimus has been used in a few young patients with hypopigmented MF. It is effective in early-stage MF, but its effectiveness is based on a few case series (140). Radiotherapy is an effective treatment, especially for tumoral lesions, but relapses frequently occur after a few months, and maintenance therapy is mandatory (141).

Systemic treatments are used mainly in advanced-stage disease, and the most used therapeutic modalities are steroids and biological response modifiers, which include interferon-alpha, retinoids (bexarotene, acitretin, isotretinoin), histone deacetylase inhibitors (romidepsin, vorinostat), brentuximab vedotin (anti-CD30), mogamulizumab (anti-CCR4), alemtuzumab (anti-CD52). Few patients receive monochemotherapy or polychemotherapy. Chemotherapy drugs have high response rates, however, the duration of response is short, with rapid recurrence. There are no standardized chemotherapy protocols, but most studies have evaluated the efficacy of gemcitabine, chlorambucil, methotrexate, pralatrexate, liposomal doxorubicin, CHOP, and CHOP-like regimens (137, 140, 142).

Allogeneic bone marrow transplantation is an effective and potentially curative treatment in advanced and refractory cases (143). Extracorporeal photopheresis is primarily indicated for erythrodermic forms (erythrodermic MF and SS) (144). Surgical excision is rarely used and is reserved for the treatment of localized nodular or tumor lesions (140).

A study comparing the therapeutic modalities used around the world has shown important differences, especially when comparing North American practices with those of other countries (European, Latin American, Asian, and Australian). Such differences occur due to the availability of different treatments and institutional experiences. Despite this variability, there are no significant differences in survival (113, 137, 141).

## 9 Conclusion

Cutaneous lymphomas are rare extranodal non-Hodgkin lymphomas. Mycosis fungoides is the most frequent cutaneous T-cell lymphoma. It is an indolent disease, with different clinical manifestations. Sézary syndrome is a rare aggressive leukemic T-cell lymphoma that presents with erythroderma, lymphadenopathy, and peripheral blood involvement. Despite the relatively good prognosis, with high survival rates in most cases, MF patients suffer from skin lesions that significantly decrease the quality of life due to concerns that the disease may progress, due to the pruritus that may be intense, and due to the social stigma caused by skin diseases. Thus, clinical recognition and correct management are essential for early diagnosis and appropriate care of these patients.

## Author contributions

DM: elaboration and final revision of the manuscript. JS: conception, elaboration, and final revision of the manuscript. All authors contributed to the article and approved the submitted version.
